# Neural basis for individual differences in the attention-enhancing effects of methylphenidate

**DOI:** 10.1073/pnas.2423785122

**Published:** 2025-03-24

**Authors:** Peter Manza, Dardo Tomasi, Şükrü Barış Demiral, Ehsan Shokri-Kojori, Christina Lildharrie, Esther Lin, Gene-Jack Wang, Nora D. Volkow

**Affiliations:** ^a^Laboratory of Neuroimaging, National Institute on Alcohol Abuse and Alcoholism, NIH, Bethesda, MD 20892; ^b^Department of Psychiatry, Kahlert Institute for Addiction Medicine, University of Maryland School of Medicine, Baltimore, MD 21201

**Keywords:** cognition, brain function, striatum, dopamine, ADHD

## Abstract

Stimulant drugs that boost dopamine, like methylphenidate (MP), enhance attention and are effective treatments for attention-deficit hyperactivity disorder (ADHD). Yet there is large individual variation in attentional capacity and response to MP. It is unclear whether this variation is driven by individual differences in relative density of dopamine receptor subtypes, magnitude of dopamine increases induced by MP, or both. Here, we extensively characterized the brain dopamine system with positron emission tomography (PET) imaging (including striatal dopamine D1 and D2/3 receptor availability and MP-induced dopamine increases) and measured attention task-evoked fMRI brain activity in two separate sessions (placebo and 60 mg oral MP; single-blind, counterbalanced) in 37 healthy adults. A network of lateral frontoparietal and visual cortices was sensitive to increasing attentional (and working memory) load, whose activity positively correlated with performance across individuals (partial r = 0.474, *P* = 0.008; controlling for age). MP-induced change in activity within this network correlated with MP-induced change in performance (partial r = 0.686, *P* < 0.001). The ratio of D1-to-D2/3 receptors in dorsomedial caudate positively correlated with baseline attentional network activity and negatively correlated with MP-induced changes in activity (all pFWE < 0.02). MP-induced changes in attentional load network activity mediated the association between D1-to-D2/3 ratio and MP-induced improvements in performance (mediation estimate = 23.20 [95%CI: −153.67 −81.79], *P* = 0.004). MP attention-boosting effects were not linked to the magnitude of striatal dopamine increases, but rather showed dependence on an individual’s baseline receptor density. Individuals with lower D1-to-D2/3 ratios tended to have lower frontoparietal activity during sustained attention and experienced greater improvement in brain function and task performance with MP.

## Significance

Stimulant medications like methylphenidate boost circulating dopamine levels and are effective treatments for attention-deficit hyperactivity disorder (ADHD), but they do not work for everyone. Here, we examined which individuals are most likely to experience the attention-enhancing benefits of methylphenidate, based on a comprehensive set of three PET and two MRI scans collected after 60 mg oral methylphenidate and placebo, in healthy adults. Counterintuitively, the magnitude of dopamine increases to a methylphenidate challenge did not predict a beneficial response. Instead, the ratio of a person’s baseline dopamine D1 versus D2 receptor availability better predicted methylphenidate-based improvements in brain function and attention. These findings describe neurobiological differences with potential relevance for stimulant drug efficacy in ADHD, though replication in clinical populations is needed.

Our ability to attend to the world around us is essential for human survival. As such, conditions with disrupted attention, such as attention-deficit hyperactivity disorder (ADHD), are associated with poor educational attainment, comorbid psychiatric illness, and a twofold increased risk in premature death (1). More broadly, “difficulty concentrating” is one of the most common symptoms across psychiatric disorders, being present in 17 different DSM-V diagnoses (2). Therefore, understanding how to enhance sustained attention is vital.

First-line therapies to enhance attention include stimulant drugs like methylphenidate (MP), which boosts synaptic concentrations of dopamine and noradrenaline by blocking their reuptake (3). There is strong scientific evidence of the central role for these neuromodulators, particularly dopamine, in MP’s efficacy. Dopamine increases produced by MP support attention via stimulation of both D1-like and D2-like receptors (D1R/D2R) in the striatum and prefrontal cortex; boosting D1R signaling enhances task-relevant representations while boosting D2R signaling suppresses task-irrelevant representations (4). MP also alters cognitive task-related brain functional activation, presumably at least in part via dopaminergic signaling. As such, MP treatment in adults with ADHD normalized aberrant brain function in the frontoparietal cortex, which is fundamental for attentional processes (5). Further, meta-analyses suggested that most studies have observed some form of “normalization” of aberrant ADHD-related activity in the striatum and prefrontal cortex during cognitive task performance with MP treatment (6, 7); recent studies continue to observe these effects (8, 9).

Thus, MP appears to improve attentional performance at least partly by enhancing signaling in dopaminergic circuits. This may be particularly beneficial in ADHD, a condition associated with aberrant levels of striatal D2/3 receptors (10), prefrontal D1 receptors (11), hypoactivity in the frontoparietal cortex during cognitive task performance (12), and disrupted functional connectivity between the dopamine-rich striatum and large swaths of the neocortex (13).

However, stimulants like MP do not work for everyone (14), and little is known about the brain basis of individual differences in the attention-enhancing effects of MP. It remains unclear whether individual differences in dopaminergic signaling and attention-related brain function could predict whether MP improves one’s attentional performance or not, and there have been recent calls to identify such brain-based markers of treatment response (15). This information would be of great clinical interest due to concerns over ambiguities in ADHD diagnosis and potential overprescription of stimulants (16), particularly since symptoms of inattention (17) and prescriptions of Schedule-II stimulants (18) have sharply increased among young and early middle age adults in the past few years.

**Fig. 1.**
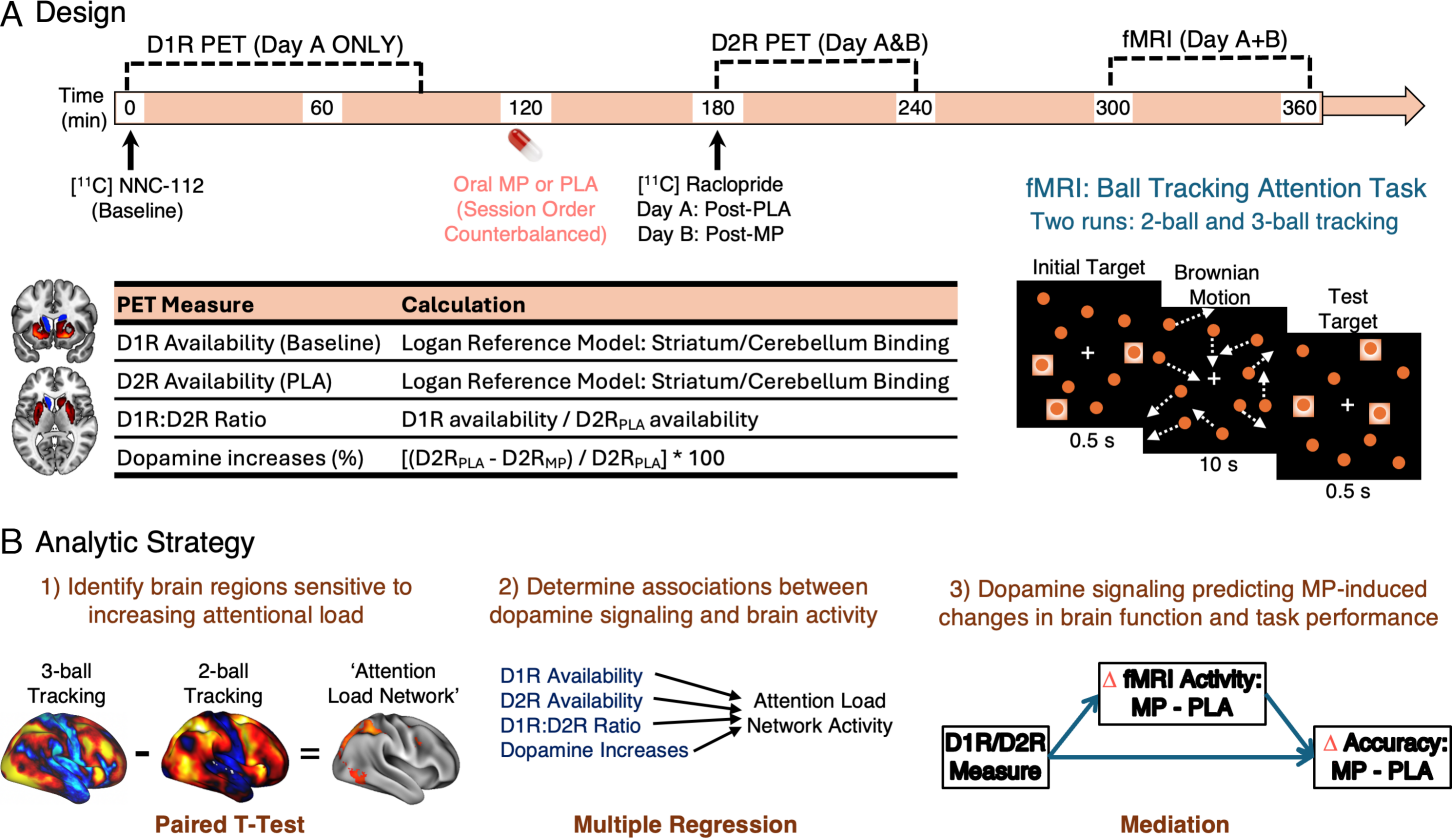
(*A*) Study design. On one day, participants underwent a [^11^C]NNC-112 scan to assess D1R availability at baseline. Then, participants took the oral medication (either 60 mg methylphenidate, MP, or placebo, PLA) and underwent a [^11^C]raclopride scan to assess dopamine D2R availability, followed by fMRI during a ball-tracking attention task. On a second day, the D2R and fMRI scans were repeated after the other oral drug was given (MP/PLA session order was counterbalanced). The fMRI scans allowed us to estimate changes in attention-related brain activity from MP-induced dopamine increases, relative to placebo. (*B*) Analytic strategy. We first identified a network of regions that was sensitive to increasing attentional load on the ball tracking task, then performed multiple regression (controlling for age) to identify significant associations between each measure of dopaminergic signaling and brain activity. Regressions were performed voxelwise over the whole striatum to identify significant clusters of PET measures that were associated with average activity across the “attention load network.” Finally, we tested whether dopamine increases might predict MP-induced changes in attention load-related brain activity, and thereby support MP-induced increases in performance (accuracy on the ball tracking task).

Here, we collected a comprehensive set of imaging measures of the brain dopamine system (including PET measurements of D1R, D2R, and MP-induced striatal dopamine increases) and fMRI paired with a task that probes both sustained attention and working memory load, collected twice (once after placebo and once after 60 mg oral MP; Fig. 1*A*). We hypothesized that greater MP-induced dopamine increases and greater D1R availability would be associated with greater task-related frontoparietal activity and better cognitive performance, since this task requires sustained attention and maintenance of visual information in working memory without switching. A body of evidence suggests that stimulating D1R-containing neurons supports maintenance of information, whereas stimulating D2R-containing neurons facilitates cognitive flexibility, enabling individuals to flexibly shift between different attention sets, tasks, or rules. Further, there appears to be a tradeoff between cognitive stability and flexibility, which may be mediated by D1R versus D2R signaling (19). Theoretically, therefore, a greater D1R-to-D2R ratio should be associated with better performance on a sustained attention task that does not have high switching demands. We previously found that the relative D1R-to-D2R ratio was much greater in association cortices than sensorimotor cortices and that across individuals, D1R-to-D2R ratio in association cortices was positively associated with spatial working memory performance (20). We sought to extend these findings by assessing the association between several striatal dopamine measures and brain function during a sustained attention fMRI task with and without a MP challenge.

## Results

**Fig. 2.**
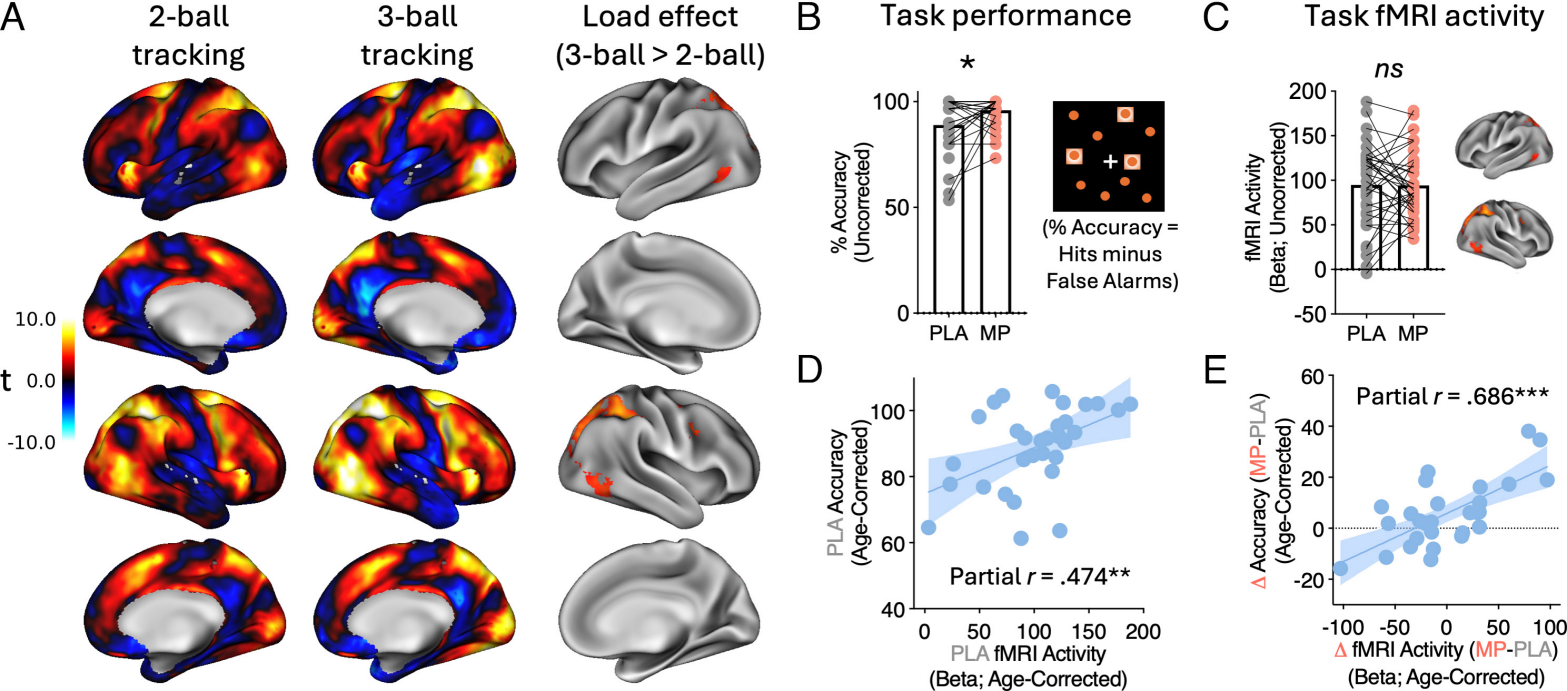
Attention fMRI task results. (*A*) Brain functional responses to attention demands. Images depict the contrast (TRACK > NO TRACK) for the lower attention load (two-ball; *Left*; unthresholded) and higher attentional load (three-ball; *Middle*; unthresholded). The attentional load effect (three-ball tracking > two-ball tracking; *Right*; significant clusters in whole-brain voxelwise analysis) yielded a network of frontoparietal and extrastriate visual cortices, as in previous work. (*B*) Task performance. Task accuracy was significantly higher in the MP session than the placebo (PLA) session. (*C*) Task fMRI activity. Brain functional activity in the ALAN of regions was not significantly different in the MP compared to PLA session. (*D*) There was a significant correlation between baseline (PLA) accuracy and baseline fMRI activity in ALAN. (*E*) MP-induced improvements in accuracy were significantly correlated with MP-induced increases in brain activation in response to attentional demands. Note: the dots in panels *D* and *E* are the residuals after correcting for age, which is why some individuals show slightly more than 100% accuracy on the scatterplot.

For an overview of the analytic strategy, see Fig. 1*B*. The primary attention task contrast (TRACK > NO TRACK) elicited positive brain activations among lateral frontal and parietal regions, as well as visual cortex, dorsal anterior cingulate cortex/presupplementary motor area, precuneus, thalamus, striatum, and cerebellum. The task elicited negative brain activations among “default mode” regions, including ventromedial, posterior cingulate, and temporal cortex, as well as motor cortex. This general pattern of activation was present for both two-ball and three-ball tracking runs (Fig. 2 *A*, *Left* and *Middle*). With increasing attentional/working memory load (three-ball tracking > two-ball tracking), there was increased activation in bilateral posterior parietal and higher-order visual cortex (MT+/V5), and right frontal eye fields (FEF). For subsequent analyses, we extracted the mean signal from this set of regions [hereafter termed “Attentional load activation network (ALAN)”] because the activation values for these clusters were all moderately-to-strongly correlated with one another (mean r = 0.64).

Participants performed generally well on the task (accuracy: placebo session = 88.92 ± 13.23 percent; MP session = 95.96 ± 6.60 percent). There were significant MP-induced increases in accuracy relative to placebo (*t*_(27)_ = 2.291, *P* = 0.030; Fig. 2*B*). However, MP did not significantly alter task-related brain activity in ALAN on average (*t*_(36)_ = 0.066, *P* = 0.95; Fig. 2*C*). Baseline (placebo session) attention–related fMRI activity was positively associated with task accuracy (partial *r* = 0.474, *P* = 0.008; Fig. 2*D*). Further, MP-induced change in activity within this network was correlated with MP-induced change in performance (partial *r* = 0.686, *P* < 0.0001; Fig. 2*E*). Thus, MP tended to increase activity in this set of regions for those with low baseline attention performance (partial *r* = −0.54, *P* = 0.002) and low baseline attention network activity (partial *r* = −0.669, *P* < 0.0001; *SI Appendix*, Fig. S1).

**Fig. 3.**
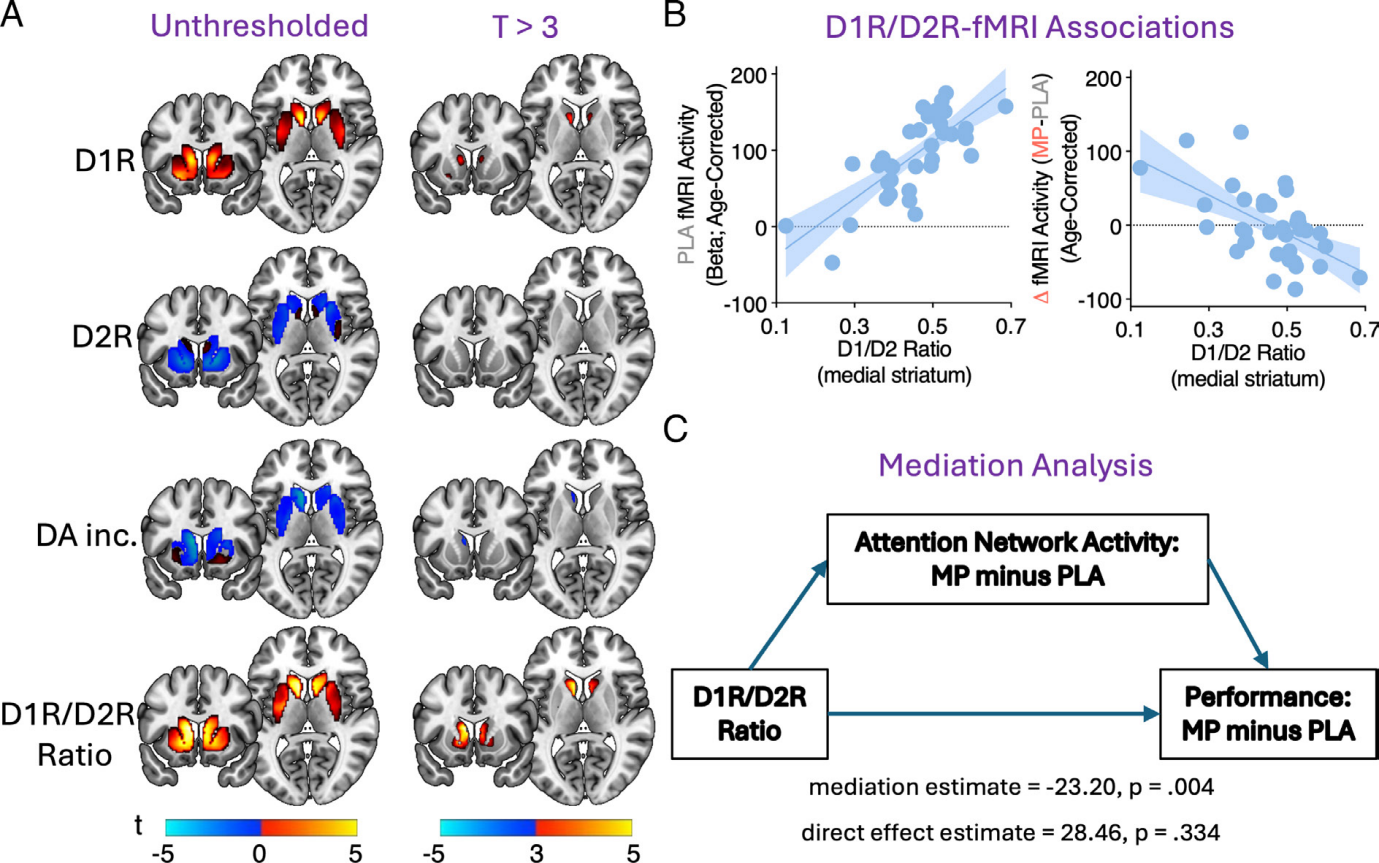
Associations between dopaminergic signaling measures and attention task fMRI. (*A*) Regressions (controlling for age) between PET-derived measures of dopaminergic function and attention task-related fMRI activity. Only the D1R/D2R ratio showed a significant association after Bonferroni correction for four comparisons. (*B*) Scatter plots depicting the associations from the cluster seen in panel *A*, *Bottom Right*. (*C*) Mediation analysis revealed that individuals with the lowest striatal D1R/D2R ratio tended to experience the greatest increases in brain activity following MP, which were associated with the largest improvements in attention task accuracy. Note: PLA = Placebo; MP = Methylphenidate; DA inc. = MP-induced dopamine increases.

We next examined associations between measures of dopaminergic function and attention-related functional activity in ALAN, again controlling for age. Regressions of D2 receptor availability and MP-induced striatal dopamine increases showed no significant associations with functional responses. However, the D1R/D2R ratio in a medial striatal cluster, encompassing the head of caudate and ventral striatum bilaterally, was strongly positively associated with PLA activity (Fig. 3 *A*, *Bottom* and Fig. 3 *B*, *Left*) and negatively associated with MP-induced change in activity in ALAN (p_FWEs_ < 0.008; Fig. 3 *B*, *Right*). This appeared to be driven primarily by D1R availability, as a similar cluster emerged in an overlapping but smaller region restricted to the head of medial caudate, that was significant in a standalone analysis (pFWE = 0.02) but would not survive Bonferroni correction for four comparisons.

Mediation analysis revealed that MP-induced changes in attention-related fMRI activity significantly mediated the association between the D1R/D2R ratio (in the significant cluster identified above) and MP-induced change in performance (mediation estimate = −23.20 [95% CI: −153.67 −81.79], *P* = 0.004; Fig. 3*C*). The direct effect was not significant (estimate = 28.46 [95% CI: −27.55 89.0]; *P* = 0.334). Thus, individuals with the lowest striatal D1R/D2R ratio tended to experience the greatest increases in ALAN activity following MP, which were associated with the largest improvements in attention task accuracy.

## Discussion

Here, we found that, among healthy adults, the individuals who most benefited from the attention-enhancing effects of MP tended to have low D1R/D2R ratio in dorsomedial caudate and low attention task-evoked activity in a network of frontal, parietal, and higher-order visual regions. The degree to which MP increased attention task activity and improved attentional performance was mediated by the relative availability of D1R/D2R in the dorsomedial caudate (but not the overall magnitude of MP-induced striatal dopamine increase). Thus, one’s baseline striatal dopamine receptor availability appears to be more predictive of attentional performance and response to MP than the magnitude of MP-induced striatal dopamine increase.

Our task fMRI analysis showed that increasing attentional (and working memory) load is associated with increased activity in the extrastriate visual cortex, posterior parietal cortex, and lateral prefrontal cortex, including the right FEF. Many studies have identified a prominent role for all of these regions in visual working memory and attention [e.g., Zarahn et al. (21)], including our prior studies with this same task (22–24). The right FEF plays an especially crucial role in this circuitry, as it has direct retinotopic projections that modulate visual cortices during attentional performance (25). Pyramidal neurons in this region encode spatial information via spiking activity tuned to a target’s location; this activity is propagated through long-range connections with posterior parietal and visual cortices to maintain information in spatial working memory (26, 27). Frontal eye field neurons with these connections preferentially exhibit D1R expression compared to D2R (28), in line with the long-observed link between prefrontal D1R signaling and working memory performance (29). Of note, MP is likely to directly affect signaling in this network, since it increases cortical dopamine (30) by blocking cortical dopamine and norepinephrine transporters (31, 32).

Our results corroborate this literature and show that in healthy adults, greater load-related activity in these regions is associated with better task performance, independent of age. Disrupting activity in this network with transcranial magnetic stimulation impairs sustained attention performance (33), whereas boosting motivation through monetary reward increases activity in these regions and improves performance (34). Here, individuals with low attention load-related activity showed the largest increase in activity and performance improvement with MP. It remains unclear if lower task activation reflects differences in regional brain metabolic activity, differences in the way these individuals understood and performed the task, or other unknown factors. A seminal FDG-PET study found global energetic deficits in ADHD, with the greatest deficits occurring in prefrontal cortices encompassing the FEF region identified here (35). We also previously showed that MP significantly reduced the regional brain metabolic increases observed with task performance (arithmetic task adjusted to an individual’s baseline 80% accuracy), an effect we interpreted to reflect MP’s improvement of brain’s efficiency (36). Interestingly, for that study, MP’s attenuation of regional brain metabolic effects were most prominent in the frontal, cingulate, and parietal cortical regions and in caudate. Our current findings are also consistent with the association between dopamine transporter availability in striatum and brain activation in posterior parietal cortex during a visual attention task (37). Though our results are based on healthy controls, neuroimaging studies in individuals with ADHD have documented functional deficits in frontal, cingulate, and parietal cortices and in caudate (38, 39), suggesting that they are clinically relevant. Our findings provide further neurobiological evidence that might underlie the “rate dependency” of MP’s behavioral effects in ADHD, which are influenced by the levels of activity observed without medication (40, 41).

The D1R/D2R ratio (primarily driven by D1R) in the dorsomedial caudate was strongly correlated with task activity in the attentional load network. These findings are consistent with numerous preclinical and clinical studies demonstrating intimate links between striatal dopaminergic function and neuronal signaling in the neocortex. For instance, selective overexpression of striatal D2R leads to GABAergic signaling deficits in layer V pyramidal neurons of mouse prefrontal cortex, providing causal evidence that the balance of striatal dopamine receptor signaling is crucial for efficient neuronal communication in extrastriatal circuits (42). In humans, higher caudate D1R was positively associated with connectivity between the right dorsolateral prefrontal cortex and right lateral parietal cortex (43, 44), and age-related reductions in caudate D1R were linked to age-related decline in working memory-related activity in these same regions (45). Further, a simultaneous PET-MRI study demonstrated strong covariation between striatal D2R expression and regional patterns of cerebral blood flow in these cortical circuits (46). Our findings linking caudate D1R/D2R signaling to the attentional load network are plausible given that they align with the anatomical organization of a known corticostriatal circuit supporting cognitive function (47). Indeed, our recent work in this same cohort showed, using cortical measures of “relative” receptor density (i.e., receptor availability normalized to the global mean), that regional estimates of D1R/D2R ratio are much higher in association cortices critical for attention task performance, as compared to primary sensorimotor cortices (20). Interestingly, in a study of 30 postmenopausal women, those with lower caudate D2R had better spatial working memory performance (48). Together with these findings, our results suggest that relatively high D1R compared to D2R signaling in caudate is a key modulator of brain function supporting spatial working memory and sustained attention.

While here we focused on striatal dopamine signaling measures, it is likely that extrastriatal dopamine receptor levels also play a key role in these findings. Prior studies found low-to-moderate correspondence between striatal versus cortical measures of D1R availability (44) as well as D2R availability (49) (most correlations: 0.3 < r < 0.5). We found a similar level of cross-regional correspondence for D1R (dorsomedial caudate versus attentional load network: partial r = 0.51, controlling for age), but not for D2R (partial r = 0.15)—and hence not for the D1R/D2R ratio (partial r = −0.27)—likely because our choice of D2R ligand, [^11^C]Raclopride, lacks sensitivity for absolute quantification of extrastriatal receptors. It is therefore unknown exactly how closely the striatal D1R/D2R ratio mirrors the prefrontal D1R/D2R ratio, and future studies using other D2R ligands, such as [^18^F]Fallypride, would be well suited to address this question.

Importantly, these findings may be specific to tasks that require high demands on information maintenance but low demands on information updating. A substantial body of theoretical and empirical (50, 51) evidence suggests that D2R signaling is crucial for working memory updating. For instance, on an n-back task, which requires continuous updating of working memory contents, one study observed positive associations between D2R in a cortico-striatal network, working memory performance, and task-related frontoparietal activity (52), whereas another study using a spatial working memory task with low updating demands found a correlation of the opposite sign (48). We therefore speculate that high levels of both D1R and D2R would support optimal performance on tasks with greater updating demands.

A key observation from our study was that individuals with the lowest D1R/D2R ratio, and relatedly, the lowest attention load-related brain activity, tended to show the biggest MP-induced improvement in attention performance. Identifying neurochemical markers of poor sustained attention and of beneficial response to MP would be of great value for improving diagnosis and personalizing the treatment of ADHD. Our results follow a line of neuroscientific study identifying several markers for positive response to stimulant drugs like MP: gray matter volume in medial prefrontal, dorsolateral prefrontal, parietal, and occipital cortices, as well as cerebellum and putamen (53, 54); MP-induced changes in blood flow in the cerebellum, dopaminergic midbrain, and dorsal prefrontal cortex encompassing frontal eye fields (55); MP-induced increases in functional connectivity between the putamen and the salience network (56); 12-wk MP treatment-related increases in the amplitude of low-frequency fluctuations in the superior parietal cortex (57); greater levels of pretreatment striatal dopamine transporter availability (58); greater cognitive control-related activity in the head of caudate (59); and higher error-related functional responses to an inhibitory control task in inferior frontal/parietal cortex and cerebellum (60). Though these studies commonly implicate cortico-striatal-cerebellar signaling, the measures of brain function were often collected using fMRI alone, for which signals are of uncertain biological origin. Here, by linking the cortical fMRI responses with dopamine receptor-specific signaling patterns, we provide neurochemical context for these individual differences in response to MP.

Surprisingly, however, the overall magnitude of dopamine increases was not significantly associated with MP-induced improvements in brain functional responses and attention task performance. At first glance, this appears to contradict our prior work showing that MP-induced ventral striatal dopamine increases were positively correlated with ADHD symptom improvement following 12 mo of treatment in adults with ADHD (61). However, our sample was characterized by healthy adults who presumably do not exhibit the same dopaminergic deficits as those with ADHD (10) and measured an acute MP challenge as opposed to chronic MP treatment. There is a well-known “inverted U” relationship between dopaminergic signaling and cognitive performance, such that moderate levels of signaling optimize performance, whereas either insufficient or excessive signaling impairs performance (62, 63). Under this model, if there is generally blunted dopaminergic signaling in ADHD, then larger dopamine increases to MP would be more likely to bring the person to optimal signaling levels and hence, dopamine increases would show a positive association with performance. However, in our study, the healthy volunteers at “baseline” are presumably already near moderate levels of dopaminergic signaling; therefore, mild to moderate increases in dopamine might not reliably be expected to produce improvements in performance, particularly under well-rested conditions.

It will be an intriguing next step to test whether the D1R/D2R associations with performance and response to MP also exist in a population with ADHD or in the early stages of Parkinson’s disease. Since our population comprised only healthy volunteers, the exact clinical relevance of our data to individuals with ADHD remains unknown. Yet they align with separate studies showing that unmedicated adults with ADHD had 1) significantly lower prefrontal and dorsal striatal D1R availability than matched controls, which correlated with symptoms of hyperactivity (11), and 2) comparable D2R availability to controls (64); combined these would manifest as an abnormally low D1R/D2R ratio in ADHD. However, another study observed lower D2R in ADHD than controls (10), so studies measuring both D1R and D2R ligands in participants with ADHD are needed to test this hypothesis. It is unclear whether differences in the D1R/D2R ratio would have relevance for the effects of other medications beneficial for ADHD (e.g., amphetamine and atomoxetine). Interestingly, both atomoxetine, a selective noradrenaline reuptake inhibitor, and MP similarly normalized frontoparietal circuits that are underrecruited in an ADHD population during an attention task (8), which may be because atomoxetine also enhances prefrontal dopamine signaling (30). Though there are comparatively fewer studies on this topic with an acute amphetamine challenge, there is evidence that, similar to MP and atomoxetine, amphetamine augments brain activation in task-relevant regions (65). Despite important differences in mechanism of action, there are many reports of similar or overlapping effects of these medications on human neurophysiology (3). Finally, it will be important to assess how sleep deprivation affects striatal D1R/D2R signaling, since we have shown in two independent studies that it reduces striatal D2R (66, 67), and because MP improves attention in healthy adults, particularly under conditions of sleep deprivation.

There are several study limitations to note. First, since [^11^C]Raclopride exhibits competitive binding with endogenous dopamine, D2R availability could be influenced not only by the quantity of unbound receptors at the cell surface but also by dopamine tone, since that determines the percentage of receptors occupied by dopamine at baseline (68). Second, some of the healthy adults here had ceiling performance (100% accuracy) on this task, suggesting the task may not have been challenging enough to see the full range in performance variation across adults. Associations between dopamine receptors and the BOLD fMRI signal are also most visible at high task demands [e.g., the three-back condition of a working memory task (52)]. Third, our study focused on striatal measures of D1R and D2R and future studies with more sensitive radiotracers are needed to evaluate how extra-striatal D1R and D2R levels influence the response to MP. Fourth, we did not have measures of the dopamine transporter, for which MP binds to directly (69). Individual differences in transporter binding efficacy and/or trafficking could explain additional variance in these findings. Fifth, nearly two-thirds of our study sample were males, and since there are documented sex differences in some components of striatal dopamine signaling (70) and small but reliable sex differences in visual attention/working memory performance (71), replication in a larger cohort with an even balance of sexes is warranted. Finally, while our PET measures were restricted to the dopamine system, MP also boosts noradrenaline signaling, particularly in extrastriatal regions, which likely plays an important role in the cognitive and brain activation enhancements from MP (72). Exploring the complex relationships between dopamine and noradrenaline transporter occupancy by MP, consequent receptor stimulation, and magnitude of MP-induced catecholamine increases alongside brain functional activation patterns would be invaluable for understanding who is most likely to benefit from MP’s attention-enhancing effects.

## Materials and Methods

Data from 37 healthy adults were included in the study (24 male, 13 female, age 22 to 64). All participants provided written informed consent, and the Institutional Review Board committee of the NIH approved the study. Participants were excluded if they had a history of substance abuse or dependence (other than nicotine) or a history of psychiatric disorder, neurological disease, medical conditions that may alter cerebral function (i.e., cardiovascular, endocrinological, oncological, or autoimmune diseases), current use of prescribed or over-the-counter psychoactive medications, and/or head trauma with loss of consciousness of >30 min. None of the healthy volunteers in this study had a past or present history of nicotine use. Portions of the PET data from this same sample were analyzed differently in prior publications (20, 70, 73, 74). However, the analyses and findings described in this manuscript are original.

### PET Acquisition and Drug Administration.

PET scans were used to measure D1R availability with [^11^C]NNC-112 and to measure D2R availability with [^11^C]Raclopride. For each individual, studies were conducted on one of two scanners: a high-resolution research tomography (HRRT) scanner (n = 17; seven females; Siemens AG; Germany) or a Biograph PET/CT scanner (n = 19; six females; Siemens AG; Germany). The use of two different scanners was necessary due to scheduling limitations at our site. The methods for correcting differences between scanners are described in the PET analysis section below. All [^11^C]NNC-112 scans were conducted at 10 AM, in a baseline state, without any drug manipulation. [^11^C]raclopride scans were conducted on two separate days: once 1 h after administration of an oral placebo pill (to assess baseline D2R availability) and once 1 h after administration of 60 mg oral MP (single blind; counterbalanced session order). Raclopride scans were conducted at the same time of day (1 PM) and in the same scanner for a given participant.

For [^11^C]NNC-112, emission scans were started immediately after an injection of ~555 MBq (mean injection activity: 15.10 ± 0.48 mCi). Twenty-one dynamic emission scans were obtained in list mode from time of injection to 90 min postinjection. For [^11^C]raclopride, emission scans were started immediately after an injection of ~370 MBq (mean injection activity: 10.04 ± 0.70 mCi). Twenty-two dynamic emission scans were obtained in list mode from time of injection to 60 min postinjection. Frames were binned as follows (in seconds): 0 30; 30 60; 60 90; 90 120; 120 150; 150 180; 180 240; 240 300; 300 360; 360 480; 480 600; 600 900; 900 1,200; 1,200 1,500; 1,500 1,800; 1,800 2,100; 2,100 2,400; 2,400 2,700; 2,700 3,000; 3,000 3,300; 3,300 3,600. One additional frame was collected in the [^11^C]raclopride scans, but it was not included in dynamic modeling because it was only 22 seconds long. Dynamic emission scan images were evaluated before analyses to ensure that motion artifacts or misplacements were not included.

### MRI Acquisition.

All subjects underwent structural and attention task functional MRI on a 3.0 T Magnetom Prisma scanner (Siemens Medical Solutions USA, Inc., Malvern, PA) with a 32-channel head coil. Subjects wore earplugs to reduce scanner noise by ~20 dB.

#### Structural MRI.

The 3D MP-RAGE (TR/TE = 2,400/2.24 ms, FA = 8 deg) and variable flip angle turbo spin-echo (Siemens SPACE; TR/TE = 3,200/564 ms) pulse sequences were used to acquire high-resolution anatomical brain images with 0.8 mm isotropic voxels field-of-view (FOV) = 240 × 256 mm, matrix = 300 × 320, and 208 sagittal slices.

#### Attention task fMRI.

To acquire fMRI time series, we used an EPI sequence with high spatiotemporal resolution, based on the sequence used for the Human Connectome Project: multiband factor = 8, anterior–posterior phase encoding, TR = 720 ms, echo time = 37 ms, flip angle = 52 deg, 72 slices with 2 mm isotropic voxels and 526 time points while the participant relaxed with their eyes open (acquisition time = 6 min 19 s per run, with two runs total). Participants performed a blocked nonverbal visual attention task that involved alternating blocks of tracking moving targets or fixating on a cross (22–24). “TRACK” epochs were composed of five 12-s-long periods (Fig. 1) in which the target balls (two or three out of 10 balls) were initially highlighted for 0.5 s. One second after the highlight disappeared, all the balls started moving randomly across the screen. Participants were asked to mentally track the moving targets while fixating on a cross at the center of the visual field. Once the all the balls stopped moving, a new set of targets was highlighted for 0.5 s. Participants were instructed to press a button if these balls matched the original set of targets. After a 1 s response window, the original target set was re-highlighted for 0.5 s to re-focus participants’ attention and the balls began to move again. During the “DO NOT TRACK” epochs, the 10 balls moved and stopped in the same manner as the “TRACK” epochs, but no balls were highlighted; during this condition, participants were instructed to fixate on the center cross and ignore the moving balls. This condition was used to control for the confounding effect of visual input activation. All subjects performed two runs of the task (two- and three-ball tracking), each one comprising three 1-min long “TRACK” and three 1-min long “DO NOT TRACK” epochs, each lasting 6 min. The task was displayed using a liquid-crystal display screen (BOLDscreen 32, Cambridge Research Systems; UK). Responses were recorded with a Lumina button box (Cedrus Corporation, CA, USA). All subjects used their right index finger for task responses. The visual attention task was presented using Visual Studio 6.0. Note that, while we have traditionally referred to this task as an “attention” task, the task manipulation alters both attentional and visual working memory loads, which cannot be disambiguated here. This is in keeping with the known substantial overlap between working memory and attentional processes in the human brain (75).

### Image Preprocessing.

FreeSurfer version 5.3.0 (http://surfer.nmr.mgh.harvard.edu) was used to automatically segment the anatomical MRI scans using the Desikan atlas (76), which provided bilateral nucleus accumbens, caudate, putamen, (which combined comprise the striatum) and cerebellum regions of interest.

We used the minimal preprocessing pipelines of the Human Connectome Project for the spatial normalization to the stereotactic MNI space of the structural MRI and subsequently the PET scans (77). Attention task fMRI time series were processed using a modified Human Connectome Project functional pipeline, which included gradient distortion correction, rigid body realignment, field map processing, and spatial normalization to the stereotactic MNI space.

Time–activity curves in voxels within the striatum were used to obtain the distribution volume ratios (DVR) using a Logan reference tissue model (78, 79) implemented in Magia (80). Striatal voxel-to-cerebellum DVR correspond to BPnd + 1, which was used to quantify D1R and D2R receptor availability. D2R images were coregistered to the high-resolution MRI T1 and T2 structural images. We also used the D2R availability estimates to compute “dopamine increases” within each voxel based on previous work:

[1] $$Dopamine\;Increases=\frac{D2R\;placebo-D2R\;methylphenidate}{D2R\;placebo},$$

Differences in geometry and PSF between cameras (PET/CT = 4 mm PSF; HRRT = 2.7 mm PSF) resulted in systematic voxelwise differences in signal intensity between PET/CT and HRRT images. To correct for these scanner-specific scaling effects and harmonize the data, we used a voxelwise approach based on grand-mean scaling. We used an updated version of the ComBat Harmonization technique implemented in the ENIGMA study (81). Originally proposed by Johnson et al. (82) and implemented in the surrogate variable analysis (sva) package in R (83), ComBat uses an Empirical Bayes framework to estimate the distribution scanner effects. It was shown to be superior to other harmonization methods for varieties of data types including DTI (84) and cortical thickness (85). We conducted ComBat separately for each tracer for the PET measure of interest (i.e., receptor availability) to harmonize the data across scanners. For [^11^C]raclopride measures, since we had placebo and MP treatments, we used drug, age, sex (male/female), and race (four groups; white, black, Asian and others) as covariates in the model. For [^11^C]NNC measures, since there was no drug manipulation, we used only age, sex, and race as covariates.

### Statistics.

#### Behavior.

The primary performance measure for the attention task is accuracy, which is calculated as the relative difference between hits and false alarms. Software issues caused problems acquiring behavioral data for several individuals: n = 6 missing placebo-session behavioral data, n = 4 missing MP-session data, and n = 1 missing both. We used all data available where possible, so that the final sample sizes for analyses were as follows: imaging n = 37; placebo accuracy n = 31; delta accuracy (MP-placebo) n = 28. To test for MP-induced changes in performance, we performed paired *t*-tests (accuracy: MP versus placebo sessions). Associations between accuracy and imaging measures are described in the following sections.

#### General lineal model.

First-level voxel-wise analyses were carried out with the general linear model in the FMRIB Software Library using the “FEAT” toolbox, estimating brain activation responses to the visual attention task for each fMRI run and subject (86). Specifically, we used a blocked analysis based on a box-car design convolved with the canonical hemodynamic response function and low-pass and high-pass (cut-off frequency: 1/256 Hz) filters.

To assess group-level brain activation patterns, we performed one-sample *t*-tests showing whole-brain voxelwise responses each for the two-ball tracking and three-ball tracking runs (contrast: TRACK > NO TRACK). Finally, to identify brain regions critical for increasing sustained attentional demand, we performed a whole-brain voxelwise paired *t*-test showing areas responsive to increasing attentional load (contrast: three-ball tracking > two-ball tracking; Fig. 1 *B*, *Left*). We then extracted the mean signal from this set of regions (termed ALAN) for the below analyses linking the fMRI data with the PET data, because the activation values for these clusters were all moderately-to-strongly correlated with one another (mean *r* = 0.64).

To describe MP-induced changes on ALAN, we performed paired *t*-tests (MP versus placebo). To characterize the associations between attention load activation and task performance, we performed linear regression models (using R’s *lm* function), with age as a covariate. We tested relationships between ALAN (placebo) and accuracy (placebo), as well as between MP-induced changes (MP minus placebo) in activation and performance.

To characterize the associations between ALAN and each of the dopaminergic measures (D1R, D2R, the D1R/D2R ratio, MP-induced dopamine increases), we performed a set of voxelwise regression analyses. We performed one regression for each PET measure, with the ALAN fMRI signal as the predictor and age as a covariate, over all striatal voxels, using the SPM12 toolbox in MATLAB.

For all voxelwise imaging analyses, the statistical significance threshold was set at *P* < 0.001 voxel-level with a *P* < 0.05 family-wise error (FWE) cluster-level correction and a minimum cluster size k = 100 voxels, in line with current reporting guidelines (87). We further Bonferroni-corrected any significant clusters from the four regression analyses (p_FWE_ < 0.05/4 = 0.0125), which may be slightly conservative because the PET measures are moderately correlated with one another (*SI Appendix*, Fig. S2).

#### Mediation.

Finally, we performed mediation analysis, hypothesizing that the MP-induced changes in ALAN would mediate the association between any significant PET measures of dopaminergic signaling (identified from the prior regression analysis) and MP-induced changes in performance. We used the “Causal Mediation Analysis” package in R (88), as in our previous work (89).

## Supplementary Material

Appendix 01 (PDF)

## Data Availability

Anonymized data used to produce the primary findings in this manuscript have been deposited in The Open Science Framework (OSF) at https://osf.io/ngx5c/ (90).
