# Supplementary material for: Neural basis for individual differences in the attention-enhancing effects of methylphenidate
Source: Proc Natl Acad Sci U S A. 2025 Mar 24;122(13):e2423785122. doi: 10.1073/pnas.2423785122 (PMC12002349; doi:10.1073/pnas.2423785122)
Supplement: Supplementary file 1 — Appendix 01 (PDF) [file pnas.2423785122.sapp.pdf]

PNAS

Supporting Information for

Neural basis for individual differences in the attention-enhancing effects of methylphenidate

Peter Manza*[1,2], Dardo Tomasi[1], Sukru Baris Demiral[1], Ehsan Shokri-Kojori[1], Christina Lildharrie[1], Esther Lin[1], Gene-Jack Wang[1], Nora D. Volkow*[1]

[1]National Institute on Alcohol Abuse and Alcoholism, National Institutes of Health, Bethesda, Maryland, USA.

[2]Kahlert Institute for Addiction Medicine, Department of Psychiatry, University of Maryland School of Medicine, Baltimore, MD

*Address correspondence to:

Peter Manza, PhD
Nora D. Volkow, MD
Email: peter.manza@som.umaryland.edu; nvolkow@nida.nih.gov

This PDF file includes:

Figures S1 to S2

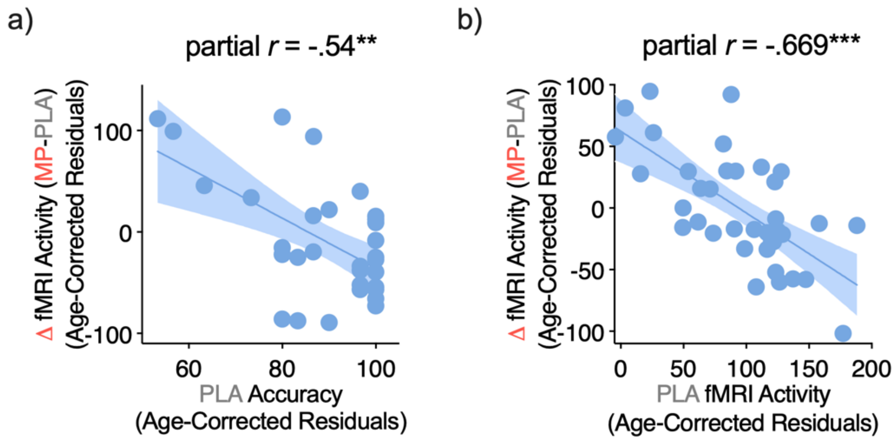

Fig. S1. Additional scatterplots depicting associations between task performance and brain activity. a) Association between baseline (placebo, PLA) attention task accuracy and methylphenidate (MP)-induced changes in brain activity, and b) Association between baseline attentional load-related brain activity and MP-induced changes in attentional load-related brain activity. Note that we cannot rule out the possibility that regression to the mean plays a role in the latter finding.

**, $p < .01$

***, $p < .001$

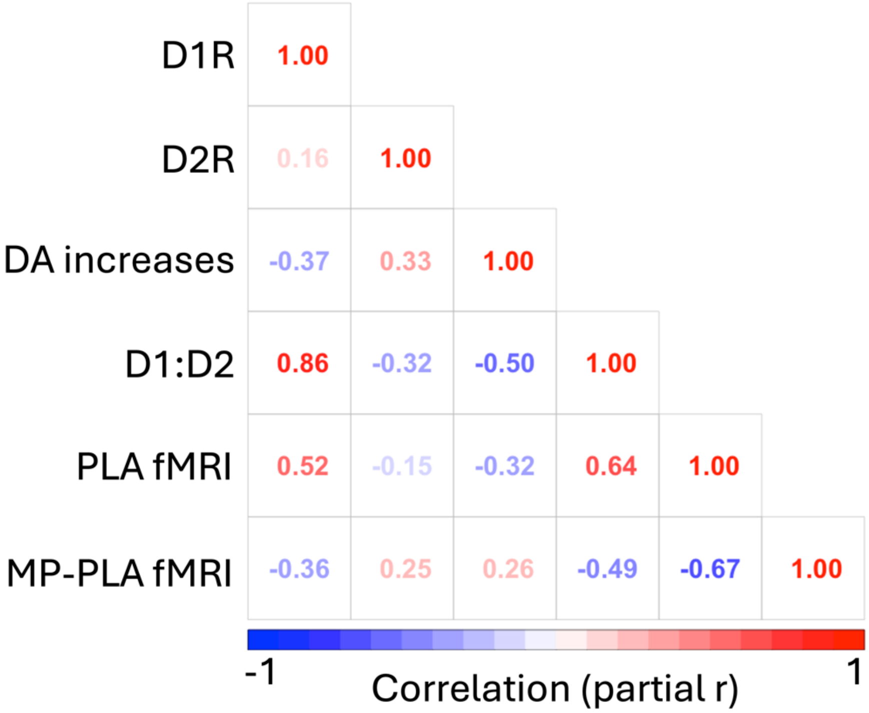

Fig. S2. Partial correlation matrix (controlling for age) between the PET and fMRI measures in the study. Note: DA = dopamine; PLA = Placebo; MP = Methylphenidate; fMRI = average attention related activity in the significant clusters identified from task fMRI analysis (shown in the main manuscript, Fig. 2).
